# Incidental diagnosis of nonfunctional bladder paraganglioma: a case report and literature review

**DOI:** 10.1186/s12894-021-00863-y

**Published:** 2021-07-08

**Authors:** Xi Tu, Neng Zhang, Xiyao Zhuang, Shulian Chen, Xu Luo

**Affiliations:** 1 Department of Urology, Affiliated Hospital of Zunyi Medical University, Zunyi, 563000 Guizhou China; 2 Department of Internal Medicine, Chengdu Shuangliu Hospital of Traditional Chinese Medicine, Chengdu, Sichuan China

**Keywords:** Paraganglioma, Bladder, Diagnosis, Treatment

## Abstract

**Background:**

Nonfunctional bladder paragangliomas is a rare urological disease. It may present clinical, radiology and pathological features similar to bladder cancer, Only scarce reports have been reported. Urologist must identify this generally benign neuroendocrine neoplasm to avoid misdiagnosis.

**Case presentation:**

A 62-year-old female presented the outpatient department of our hospital with the symptoms of stomachache, frequent micturition, and urination pain for 20 days. Diagnosed with high blood pressure 1 year ago, administered Amlodipine besylate tablets 5 mg po qd occasionally, did not check blood pressure; denied any tumor observation in the family history. Color ultrasound of the urinary system showed a 38 mm × 34 mm hypoechoic mass on the right side of the bladder, CDFI: in the masses, blood supply was sufficient. Cystoscope showed bladder occupying lesion. Biopsy diagnosis: papillary polypoid cystitis was suspected as a malignant change (Fig. 3a). Then, the patient was admitted to our urological department. Further, computer tomography urography considered bladder cancer. Cystoscopy and biopsy failed to define the nature of the lesions in our outpatient department, which prompted a transurethral resection of the bladder tumor. histopathological and immunohistochemical results were diagnosed as bladder paragangliomas. For the reason, the tumor was removed by partial resection of the bladder. The postoperative recovery and follow-up were uneventful.

**Conclusions:**

Nonfunctional bladder paragangliomas are occasionally found on imaging studies with the symptoms of urinary tract infection or/and intermittent painless hematuria. It may present clinical, radiology and pathological features similar to bladder cancer, so knowledge of this generally benign neuroendocrine neoplasm is of great importance to avoid misdiagnosis. It should be accompanied by the clinical and pathological characteristics of the patient and image changes. Partial resection of the bladder can effectively treat this disease.

## Background

Paraganglioma of the urinary bladder (PUB) is a rare disease, accounting for 0.06% of all bladder tumors [1]. The PUB symptoms are correlated with over secretion of catecholamine or tumor impact. It is classified into functional and non-functional. Functional PUB usually exhibits symptoms such as headache, syncope, palpitation, and sweating. However, 10–15% of these tumors are nonfunctional and in 10%, hormone activity does not manifest clinically [2]. Non-functional PUB might have symptoms such as frequency and urgency of urination, intermittent painless whole course gross hematuria, and lumbar discomfort. In addition, histological characteristics such as the death of focal transparent cells and burnt muscle infiltration might be easily diagnosed as bladder cancer [3]. The present study reported a case of a female patient who underwent bladder tumor resections two times due to the confusion of bladder cancer and whose histopathological and immunohistochemical results confirmed paraganglioma.

## Case presentation

### Chief complaints

A 62-year-old female presented the outpatient department of our hospital with the symptoms of stomachache, frequent micturition, and urination pain.

### History of present illness

Patient’s symptoms started 20 days ago, She had no symptoms such as lumbago, dizziness, headache, palpitation, chest tightness, nausea, vomiting, fever, chills, or other symptoms.

### History of past illness

The patient was diagnosed with high blood pressure 1 year ago, administered Amlodipine besylate tablets 5 mg po qd occasionally, did not check blood pressure; terminal gross hematuria was observed once 4 months ago, the symptom disappeared, and no treatment was administered; denied any tumor observation in the family history.

### Physical examination

After hospitalization, the patient’s temperature was 36.5 ℃, 80 pulses/min, 20 breathing times/min, blood pressure was 159/108 mmHg and oxygen saturation in room air was 98%. no bulge, pressure pain or percussion pain in either of the kidney area; no pressure or percussion pain in both ways of the ureteral region; no bulge in the bladder area but light pressure-induced pain.

### Supplementary examination

**Fig. 1:**
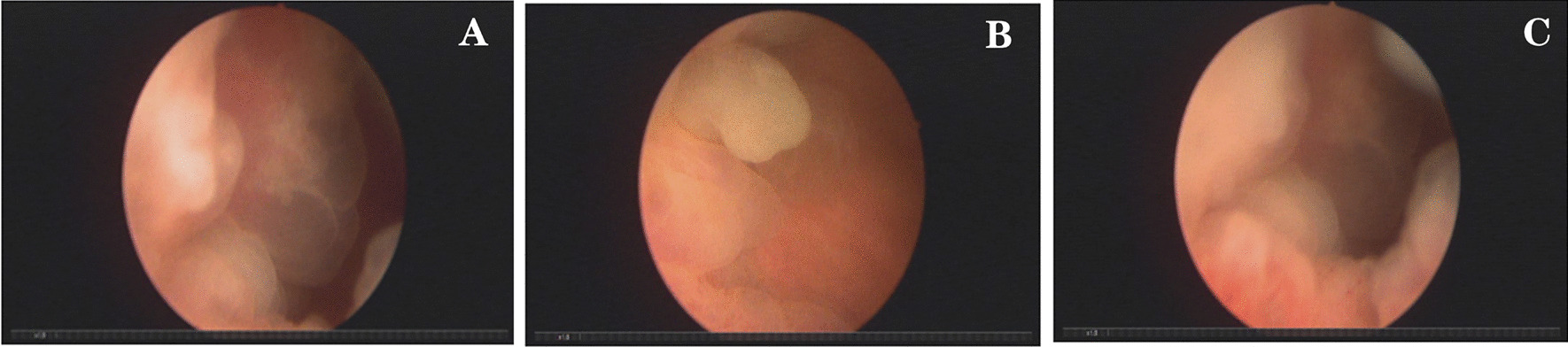
Cystoscope. **a**, **b** Large tumor was observed in the right side of the bladder; a large number of growing follicles were observed; dead tissue and broad-based were seen; **c** the bottom of the tumor continued to the entrance of the right side

**Fig. 2:**
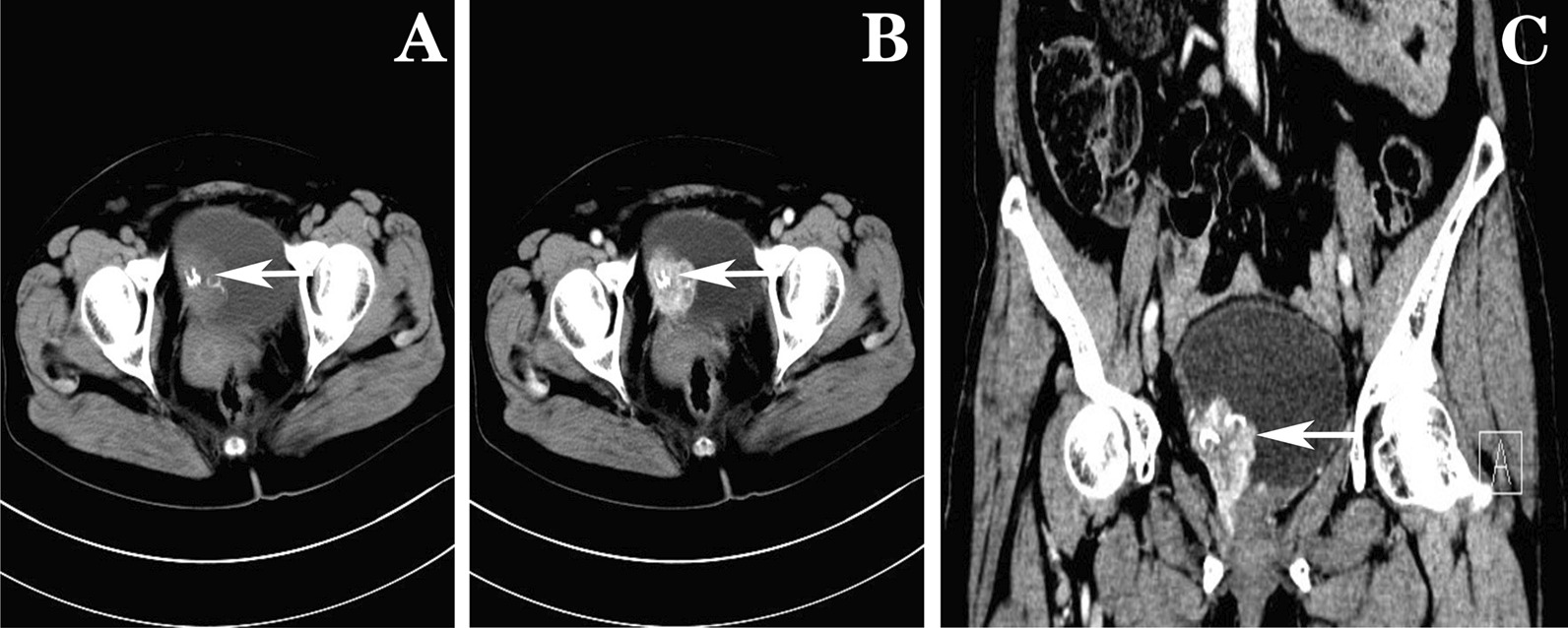
CTU. **a** This images showed 34 mm × 37 mm low-density mass on the right side of the bladder with clear edges, and Calcium density shadow was seen inside; **b**, **c** the enhancement scanning was uneven

**Fig. 3:**
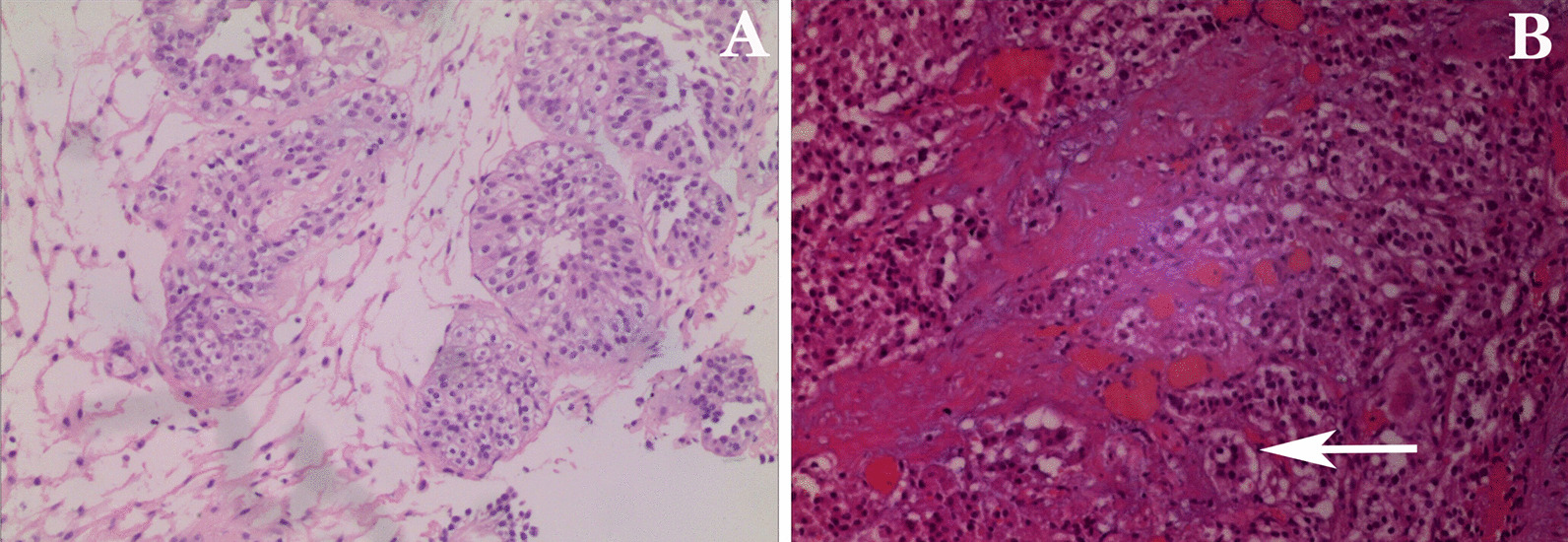
Pathology. **a** This image showed papillary polypoid cystitis; **b** arrow showed tumor cells were arranged in small nests, the interstitial blood sinuses were also abundant

Fasting venous blood glucose was 8.34 mmol/L, and other indexes were normal. Chest X-ray showed mild interstitial changes in both lungs, ECG: 102 times/min. Color ultrasound of the urinary system showed a 38 mm × 34 mm hypoechoic mass on the right side of the bladder with an uneven surface and a large bottom; multiple strong echo masses were observed. CDFI: in the masses, blood supply was sufficient. Cystoscope showed bladder occupying lesion (Fig. 1). Biopsy diagnosis: papillary polypoid cystitis was suspected as a malignant change (Fig. 3a). Computer tomography urography(CTU) considered bladder cancer (Fig. 2).

### Diagnosis and treatment process

Cystoscopy and biopsy failed to define the nature of the lesions. the urethral bladder tumor biopsy was performed. During the operation, the right bladder wall adjacent to the neck of the bladder showed vegetations with a size of about 4.5 cm × 4.5 cm, with a broad base, easy bleeding, and local surface tissue ulceration. The tumor was found to be hard and calcified during electrotomy. The histopathological studies revealed a large number of tumor cells proliferated with abundant cytoplasm, tumor cells were arranged in small nests or strips, and the blood sinusoids between tumor cells were abundant (Fig. 3b). The immunohistochemical study was positive for the expression for CD56, chromogranin A, neuron specific enolase and synaptophysin. Immunostaining for the polyclonal S-100 protein showed a sustentacular pattern, thereby confirming paraganglioma. Therefore, another operation was needed. The patient regularly administered phenoxybenzamine 10 mg po tid and metformin tablets 0.5 g po tid for 2 weeks. Before the operation, laboratory workup revealed elevated plasma renin activity to 8642.76 pg/mL, elevated angiotensin I to 16113.5 pg/mL, and an elevated angiotensin II level of 252.91 pg/mL. Partial resection of the bladder was conducted, and the focus was removed. After the operation, the patient stopped using phenoxybenzamine and continued with metformin tablets. Consecutively, blood pressure and blood sugar were monitored. the plasma renin activity, angiotensin I and angiotensin II were within normal range (56.83 pg/mL, 447.46 pg/mL, 79.04 pg/mL, respectively). When the patient was ready to leave the hospital, her blood pressure was 128/86 mmHg, the blood sugar was 7.0–9.4 mmol/L, and the symptoms had improved.

### Follow-up

Three and six months after the operation, no discomfort was reported. Blood sugar and blood pressure were at normal levels. No obvious abnormality was detected by cystoscopy. However, The patient was advised regular reexamination and lifelong follow-up, with respect to blood pressure, blood glucose, urinary catecholamine, ultrasound, CT, and cystoscopy.

## Discussion and conclusion

PUB mainly occurs in young Caucasians, aged 20-40-years-old, with a male to female ratio of 1:3, mostly single, and common in the anterior wall, posterior wall, and top of the bladder. The triangle was rare [1, 4]. The cause of the disease was unknown. In the case of a familial paraganglioma, it may be related to the mutation in the succinate dehydrogenase coding gene [5]. Most of the PUB was located in the submucous or muscular layer of the bladder wall; about 37% could penetrate the whole bladder wall or the pelvic wall. In this case, the lesion is mainly located on the right lower wall of the bladder, and the tumor invades the lower part of the right ureter, causing the upper urinary tract to dilate and accumulate. The patient only showed symptoms of abdominal pain, frequent urination, and painful urination, and did not show symptoms of syncope, headache, palpitations, or sweating caused by increased catecholamine secretion. So it was considered non-functional bladder paraganglioma.

Color ultrasound of the urinary system is the first line of investigative modality to detect the silent as well as functioning ectopic lesions. Doppler demonstrates the vascular nature of these tumors, which was also observed in our case. the tumor showed a low-density shadow mass with clear edge, calcium density shadow and uneven enhancement on CT. On magnetic resonance imaging (MRI), these tumors are markedly hyperintense on T2-weighted (T2W) images. In addition to the monitoring of catecholamine or its metabolites, echocardiography, CT, magnetic resonance imaging (MRI), and radiotracer molecular imaging in the clinical suspicion of PUB is essential. The functional imaging technology of the tracer molecular may confirm that the tumor is paraganglioma (such as ^123/131^I-MIBG, ^18^ F-FDA, ^18^ F-FDG, ^18^ F-FDOPA, ^11^ C-ephedrine, ^11^ C-hydroxyethyl ephedra alkali.) [5]. The histological characteristics of paraganglioma are similar to those of bladder cancer. Paragangliomas commonly arise in the deep layers of the bladder wall, so when they develop they affect their own muscle layer, making it even more difficult to make the differential diagnosis with a tendency to diagnose urothelial carcinoma. Histologically, the paraganglioma is usually composed of cells with a “zellballen” pattern with abundant eosinophilic or amphophilic cytoplasm divided by delicate vascular stroma [3]. Biopsy of tumor tissue will confirm diagnosis with diffuse, strong positivity for neuron-specific enolase, synaptophysin, and/or chromogranin [6]. The case in this report was considered to be bladder cancer before the operation because the patient had no typical clinical symptoms related to hypersecretion of catecholamines. In addition, due to the lack of experience in diagnosis and treatment and the lack of in-depth combination with the patient’s history of hypertension and high blood sugar after admission, the correct diagnosis was not made initially. In order to clarify the nature of the tumor, transurethral resection of the bladder tumor was carried out. histopathological and immunohistochemical results confirmed paraganglioma.

The PUB is not sensitive to radiotherapy and chemotherapy. The most common treatment is partial cystectomy. Other treatments include transurethral cystectomy and radical cystectomy. The symptoms can be controlled, and low incidence can be achieved by surgical resection. Adequate preoperative preparation can significantly reduce the risk of hypertension and the hazards of operation. In this case, the patient had been regularly taking phenoxybenzamine for 2 weeks. Her blood pressure was stable before, during, and after the operation. The surgical treatment of metastatic pheochromocytoma is rarely effective. Radical bladder surgery, bilateral pelvic lymphadenectomy, radiotherapy, and chemotherapy are feasible for the cases with wide pathological range, impossible bladder preservation, and lymph node metastasis. Reportedly, 76% of the patients with paraganglioma received > 40 Gy of external irradiation, and their clinical symptoms were significantly relieved for at least 1 year or until death [7]. Patients who did not benefit from the operation or local radiotherapy could choose ^131^I-MIBG and cytotoxic chemotherapy. The study found that MIBG treatment was beneficial to 67% of the patients’ biochemical reactions, and 89% of the patients’ symptoms were improved [8]. However, the combination of external irradiation and ^131^I-MIBG has a good effect on the local control and treatment of malignant paraganglioma [7]. Cytotoxic chemotherapy is feasible for patients with paragangliomas that cannot be resected as they progress rapidly or exhibit marked bone metastasis. The most widely used chemotherapy regimens include CVD (cyclophosphamide, vincristine, and dacarbazine). The survival rate of patients with malignant paragangliomas is variable. According to the study population, the overall 5-year survival rate is 35–60% [6].

Nonfunctional bladder paragangliomas are occasionally found on imaging studies with the symptoms of urinary tract infection or/and intermittent painless hematuria. It may present clinical, radiology and pathological features similar to bladder cancer, so knowledge of this generally benign neuroendocrine neoplasm is of great importance to avoid misdiagnosis. In our study, the patient underwent open surgery to remove the tumor completely. The prognosis was good.

## Data Availability

The datasets used and/or analysed during the current study are available from the corresponding author on reasonable request.

## Abbreviations

CT: Computed tomography
CDFI: Color doplor flow image
ECG: Electrocardiograph
PUB: Paraganglioma of the urinary bladder
CTU: Computer tomography urography
MIBG: Metaiodobenzylguanidine
MRI: Magnetic resonance imaging
18F-FDA: 18 F-fluorodopamine
18F-FDG: 18 F-fluoro-2-deoxy-D-glucose
18F-FDOPA: 18 F-fluoro-dihydroxyphenylalanine
I-MIBG: Iodine-labeled meta-iodobenzylguanidine
CVD: Cyclophosphamide, vincristine, and dacarbazine
